# Supplementary material for: Slow oscillation–spindle coupling strength predicts real-life gross-motor learning in adolescents and adults
Source: eLife. 2022 Feb 21;11:e66761. doi: 10.7554/eLife.66761 (PMC8860438; doi:10.7554/eLife.66761)
Supplement: Supplementary file 1. [file elife-66761-supp1.docx]

**SUPPLEMENTARY FILE**

**Table 1 related to Figure 1**. Sleep architecture (mean ± standard deviation) for the adaptation and learning night collapsed across both age groups. Nights were compared using paired t-tests

| All | Adaptation night | | | Learning night | | | p-value |
| --- | --- | --- | --- | --- | --- | --- | --- |
| Time in bed (min) | 507.292 | ± | 29.981 | 507.453 | ± | 29.280 | 0.929 |
| Total sleep time (min) | 471.283 | ± | 40.029 | 483.915 | ± | 29.737 | **0.001** |
| Sleep onset latency to NREM2 (min) | 23.387 | ± | 19.042 | 19.585 | ± | 14.034 | 0.089 |
| Sleep efficiency (%) | 92.935 | ± | 5.095 | 95.524 | ± | 2.802 | **<0.001** |
| NREM1 (min) | 41.849 | ± | 30.899 | 34.877 | ± | 24.615 | **0.004** |
| NREM1 (%) | 9.240 | ± | 7.279 | 7.442 | ± | 5.564 | **0.001** |
| NREM2 (min) | 153.698 | ± | 49.949 | 156.972 | ± | 54.46 | 0.386 |
| NREM2 (%) | 33.100 | ± | 11.622 | 32.936 | ± | 12.429 | 0.826 |
| NREM3 (min) | 212.717 | ± | 98.675 | 220.802 | ± | 99.815 | 0.086 |
| NREM3 (%) | 44.163 | ± | 18.537 | 44.829 | ± | 18.604 | 0.452 |
| REM (min) | 63.019 | ± | 21.108 | 71.264 | ± | 24.536 | **0.007** |
| REM (%) | 13.498 | ± | 4.587 | 14.793 | ± | 5.168 | **0.033** |
| Wake after sleep onset (min) | 17.377 | ± | 16.294 | 8.689 | ± | 6.708 | **<0.001** |

**Table 2 related to Figure 1**. Summary of sleep architecture and SO/spindle event descriptive measures (at electrode C4) of adolescents and adults across the whole sample (mean ± standard deviation) in the learning night. Independent t-tests were used for comparisons

| All | Adolescents | | | Adults | | | p-value |
| --- | --- | --- | --- | --- | --- | --- | --- |
| Time in bed (min) | 531.679 | ± | 18.773 | 480.708 | ± | 2.445 | **<0.001** |
| Total sleep time (min) | 506.589 | ± | 20.402 | 458.438 | ± | 13.550 | **<0.001** |
| Sleep onset latency to NREM2 (min) | 18.821 | ± | 15.632 | 20.813 | ± | 12.381 | 0.617 |
| Sleep efficiency (%) | 95.520 | ± | 2.843 | 95.434 | ± | 2.834 | 0.914 |
| NREM1 (min) | 17.839 | ± | 8.592 | 55.167 | ± | 22.27 | **<0.001** |
| NREM1 (%) | 3.522 | ± | 1.686 | 12.100 | ± | 5.012 | **<0.001** |
| NREM2 (min) | 124.821 | ± | 49.137 | 196.104 | ± | 30.804 | **<0.001** |
| NREM2 (%) | 24.728 | ± | 9.985 | 42.817 | ± | 6.783 | **<0.001** |
| NREM3 (min) | 297.482 | ± | 65.336 | 130.792 | ± | 43.521 | **<0.001** |
| NREM3 (%) | 58.660 | ± | 12.476 | 28.444 | ± | 9.230 | **<0.001** |
| REM (min) | 66.446 | ± | 27.011 | 76.375 | ± | 21.037 | 0.151 |
| REM (%) | 13.090 | ± | 5.265 | 16.640 | ± | 4.502 | **0.013** |
| Wake after sleep onset (min) | 7.661 | ± | 6.285 | 10.125 | ± | 7.108 | 0.191 |
| SO (N) | 3499.107 | ± | 340.288 | 1855.280 | ± | 632.753 | **<0.001** |
| SO density (N/NREM3 epoch) | 6.141 | ± | 1.347 | 7.215 | ± | 1.997 | **0.025** |
| Spindle number (N) | 3506.571 | ± | 742.618 | 1439.080 | ± | 580.892 | **<0.001** |
| Spindle density (N/NREM3 epoch) | 5.935 | ± | 0.603 | 5.383 | ± | 1.159 | **0.032** |
| Event co-occurrence (N) | 1623.357 | ± | 295.338 | 753.720 | ± | 299.714 | **<0.001** |
| Event co-occurrence (%) | 47.359 | ± | 8.030 | 53.745 | ± | 11.424 | **0.021** |
| Coupling strength | 0.071 | ± | 0.038 | 0.133 | ± | 0.0510 | **<0.001** |

**Table 3 related to Figure 2D.** Mixed ANOVA Output comparing juggling learning curves pre- and post retention interval 1 between the condition groups and age groups

| Effect | df | F-statistic | p-value | Effect size (η^2^) |
| --- | --- | --- | --- | --- |
| Performance test (pre-, post retention) | 1 | 1.812 | 0.183 | 0.027 |
| Condition group (sleep first, wake first) | 1 | 0.082 | 0.775 | 0.001 |
| Age group (adolescents, adults) | 1 | 0.992 | 0.323 | 0.015 |
| Condition group*Age group | 1 | 0.238 | 0.627 | 0.004 |
| Performance test*Condition group | 1 | 4.868 | **0.031** | 0.070 |
| Performance test*Age group | 1 | 0.026 | 0.873 | < 0.001 |
| Performance test*Condition group*Age group | 1 | 0.093 | 0.761 | 0.001 |
| Error | 65 |  |  |  |

**Table 4 related to Figure 2E.** Mixed ANOVA Output comparing juggling task proficiency pre- and post retention interval 1 between the condition groups and age groups

| Effect | df | F-statistic | p-value | Effect size (η^2^) |
| --- | --- | --- | --- | --- |
| Performance test (pre-, post retention) | 1 | 0.153 | 0.697 | 0.002 |
| Condition group (sleep first, wake first) | 1 | 0.001 | 0.972 | < 0.001 |
| Age group (adolescents, adults) | 1 | 2.338 | 0.131 | 0.035 |
| Condition group*Age group | 1 | 5.210 | **0.026** | 0.074 |
| Performance test*Condition group | 1 | 1.882 | 0.175 | 0.028 |
| Performance test*Age group | 1 | 0.009 | 0.925 | < 0.001 |
| Performance test*Condition group*Age group | 1 | 0.026 | 0.873 | < 0.001 |
| Error | 65 |  |  |  |

**Table 5 related to Figure 2**. Summary of linear mixed models for predicting learning curve, PVT mean reaction time and task proficiency separately across all performance tests and for the first performance test only using the structure ~Age group + Time of day + (1|Subjects)

|  | **Modeled** | **Parameter** | **Beta** | **T-statistic** | **df** | **p-value** | **Lower-95 CI** | **Upper-95 CI** | **Random effects (SD)** |
| --- | --- | --- | --- | --- | --- | --- | --- | --- | --- |
| (**A**) | Learning curve (all performance tests) | Intercept | 2.922 | 6.832 | 202 | < 0.001 | 2.079 | 3.766 | 1.169 |
|  |  | Age group adult | -0.991 | -2.043 | 202 | 0.042 | -1.948 | -0.035 |  |
|  |  | Time of day evening | -1.129 | -2.885 | 202 | 0.004 | -1.901 | -0.357 |  |
|  |  |  |  |  |  |  |  |  |  |
| (**B**) | Learning curve (first performance test) | Intercept | 2.553 | 4.544 | 66 | < 0.001 | 1.431 | 3.675 | 1.648 |
|  |  | Age group adult | -0.540 | -0.945 | 66 | 0.348 | -1.681 | 0.601 |  |
|  |  | Time of day evening | -1.294 | -2.251 | 66 | 0.028 | -2.442 | -0.146 |  |
|  |  |  |  |  |  |  |  |  |  |
| **(C**) | Task proficiency (all performance tests) | Intercept | 14.747 | 5.407 | 202 | < 0.001 | 9.369 | 20.124 | 12.788 |
|  |  | Age group adult | 8.082 | 2.371 | 202 | 0.019 | 1.36 | 14.804 |  |
|  |  | Time of day evening | 2.7467 | 1.999 | 202 | 0.047 | 0.037 | 5.456 |  |
|  |  |  |  |  |  |  |  |  |  |
| (**D**) | Task proficiency (first performance test) | Intercept | 12.804 | 3.791 | 66 | < 0.001 | 6.060 | 19.547 | 9.905 |
|  |  | Age group adult | 6.285 | 1.830 | 66 | 0.072 | -0.571 | 13.141 |  |
|  |  | Time of day evening | 3.470 | 1.004 | 66 | 0.318 | -3.428 | 10.369 |  |
|  |  |  |  |  |  |  |  |  |  |
| (**E**) | PVT mean reaction time (all performance tests) | Intercept | 326.29 | 43.481 | 202 | < 0.001 | 311.5 | 341.09 | 32.550 |
|  |  | Age group adult | -23.762 | -2.585 | 202 | 0.010 | -41.886 | -5.639 |  |
|  |  | Time of day evening | -1.107 | -0.238 | 202 | 0.812 | -10.29 | 8.077 |  |
|  |  |  |  |  |  |  |  |  |  |
| (**F**) | PVT mean reaction time (first performance test) | Intercept | 311.37 | 31.469 | 66 | < 0.001 | 291.61 | 331.12 | 29.015 |
|  |  | Age group adult | -14.209 | -1.413 | 66 | 0.163 | -34.294 | 5.876 |  |
|  |  | Time of day evening | 9.622 | 0.951 | 66 | 0.345 | -10.586 | 29.831 |  |

Table note: linear mixed model we computed with age group (adolescents, adults) and time of day (i.e. performance test in the morning or evening) as fixed effects and subjects as random effects with the fitlme.m matlab function using maximum likelihood estimation. We used reference dummy coding, where the coefficient of the first category is set to 0 (i.e. fixed effect of age group is referenced to adolescents whereas the Time of day fixed effect is referenced to performance tests in the morning).

**Table 6 related to Figure 2**. Summary of linear mixed models for predicting learning curve and task proficiency separately across all performance tests using the structure ~Time of day + Sleep after learning + (1|Subjects). Sleep after learning fixed effect contains performance test 2 for the sleep first group and performance test 3 for the wake first group.

|  | **Modeled** | **Parameter** | **Beta** | **T-statistic** | **df** | **p-value** | **Lower-95 CI** | **Upper-95 CI** | **Random effects (SD)** |
| --- | --- | --- | --- | --- | --- | --- | --- | --- | --- |
| (**A**) | Learning curve (all performance tests) | Intercept | 2.206 | 3.863 | 202 | < 0.001 | 1.080 | 3.333 | 1.272 |
|  |  | Time of day evening | -1.008 | -1.625 | 202 | 0.106 | -2.231 | 0.215 |  |
|  |  | Sleep after learning | 0.172 | 0.268 | 202 | 0. 789 | -1.093 | 1.437 |  |
|  |  |  |  |  |  |  |  |  |  |
| (**B**) | Task proficiency (all performance tests) | Intercept | 16.677 | 6.540 | 202 | < 0.001 | 11.648 | 21.705 | 13.391 |
|  |  | Time of day evening | 5.751 | 2.252 | 202 | 0.011 | 1.310 | 10.192 |  |
|  |  | Sleep after learning | 3.795 | 1.672 | 202 | 0.096 | -0.680 | 8.271 |  |
|  |  |  |  |  |  |  |  |  |  |

**Table 7 related to Figure 3DE, Figure 3 – figure supplement 3 & 4**. Summary of sleep architecture and SO/spindle event descriptive measures (at electrode C4) of adolescents and adults in the sleep first group (mean ± standard deviation) in the learning night. Independent t-tests were used for comparisons

| Sleep first | Adolescents | | | Adults | | | p-value |
| --- | --- | --- | --- | --- | --- | --- | --- |
| Time in bed (min) | 530.118 | ± | 17.407 | 480.708 | ± | 2.445 | **<0.001** |
| Total sleep time (min) | 502.059 | ± | 19.204 | 458.438 | ± | 13.550 | **<0.001** |
| Sleep onset latency to NREM2 (min) | 21.794 | ± | 17.474 | 20.813 | ± | 12.381 | 0.834 |
| Sleep efficiency (%) | 94.771 | ± | 3.128 | 95.434 | ± | 2.834 | 0.484 |
| NREM1 (min) | 16.088 | ± | 9.805 | 55.167 | ± | 22.27 | **<0.001** |
| NREM1 (%) | 3.206 | ± | 1.930 | 12.100 | ± | 5.012 | **<0.001** |
| NREM2 (min) | 115.647 | ± | 54.532 | 196.104 | ± | 30.804 | **<0.001** |
| NREM2 (%) | 23.137 | ± | 11.053 | 42.817 | ± | 6.783 | **<0.001** |
| NREM3 (min) | 296.147 | ± | 71.121 | 130.792 | ± | 43.521 | **<0.001** |
| NREM3 (%) | 58.942 | ± | 13.758 | 28.444 | ± | 9.230 | **<0.001** |
| REM (min) | 74.176 | ± | 25.138 | 76.375 | ± | 21.037 | 0.763 |
| REM (%) | 14.712 | ± | 4.891 | 16.640 | ± | 4.502 | 0.200 |
| Wake after sleep onset (min) | 8.294 | ± | 7.782 | 10.125 | ± | 7.108 | 0.439 |
| SO (N) | 3498.118 | ± | 318.304 | 1855.280 | ± | 632.753 | **<0.001** |
| SO density (N/NREM3 epoch) | 6.226 | ± | 1.508 | 7.215 | ± | 1.997 | 0.091 |
| Spindle number (N) | 3477.294 | ± | 819.27 | 1439.080 | ± | 580.892 | **<0.001** |
| Spindle density (N/NREM3 epoch) | 5.915 | ± | 0.669 | 5.383 | ± | 1.159 | 0.096 |
| Event co-occurrence (N) | 1617.941 | ± | 337.016 | 753.720 | ± | 299.714 | **<0.001** |
| Event co-occurrence (%) | 47.709 | ± | 8.546 | 53.745 | ± | 11.424 | 0.071 |
| Coupling strength | 0.067 | ± | 0.039 | 0.133 | ± | 0.0510 | **<0.001** |

**Table 8 related to Figure 3 – figure supplement 2AB**. Summary of sleep architecture and SO/spindle event descriptive measures (at electrode C4) of adolescents in the sleep first and wake first group (mean ± standard deviation). Independent t-tests were used for comparisons

| Adolescents | Sleep first | | | Wake first | | | p-value |
| --- | --- | --- | --- | --- | --- | --- | --- |
| Time in bed (min) | 530.118 | ± | 17.407 | 534.091 | ± | 21.359 | 0.594 |
| Total sleep time (min) | 502.059 | ± | 19.204 | 513.591 | ± | 21.095 | 0.147 |
| Sleep onset latency to NREM2 (min) | 21.794 | ± | 17.474 | 14.227 | ± | 11.531 | 0.217 |
| Sleep efficiency (%) | 94.771 | ± | 3.128 | 96.677 | ± | 1.937 | 0.083 |
| NREM1 (min) | 16.088 | ± | 9.805 | 20.545 | ± | 5.677 | 0.185 |
| NREM1 (%) | 3.206 | ± | 1.930 | 4.009 | ± | 1.136 | 0.225 |
| NREM2 (min) | 115.647 | ± | 54.532 | 139.000 | ± | 37.374 | 0.226 |
| NREM2 (%) | 23.137 | ± | 11.053 | 27.187 | ± | 7.921 | 0.303 |
| NREM3 (min) | 296.147 | ± | 71.121 | 299.545 | ± | 58.524 | 0.896 |
| NREM3 (%) | 58.942 | ± | 13.758 | 58.224 | ± | 10.818 | 0.885 |
| REM (min) | 74.176 | ± | 25.138 | 54.500 | ± | 26.462 | 0.058 |
| REM (%) | 14.712 | ± | 4.891 | 10.583 | ± | 5.018 | **0.040** |
| Wake after sleep onset (min) | 8.294 | ± | 7.782 | 6.682 | ± | 2.831 | 0.518 |
| SO (N) | 3498.118 | ± | 318.304 | 3500.636 | ± | 387.991 | 0.985 |
| SO density (N/NREM3 epoch) | 6.226 | ± | 1.508 | 6.010 | ± | 1.109 | 0.687 |
| Spindle number (N) | 3477.294 | ± | 819.27 | 3551.818 | ± | 641.377 | 0.801 |
| Spindle density (N/NREM3 epoch) | 5.915 | ± | 0.669 | 5.965 | ± | 0.512 | 0.835 |
| Event co-occurrence (N) | 1617.941 | ± | 337.016 | 1631.727 | ± | 231.629 | 0.907 |
| Event co-occurrence (%) | 47.709 | ± | 8.546 | 46.818 | ± | 7.531 | 0.780 |
| Coupling strength | 0.067 | ± | 0.039 | 0.077 | ± | 0.037 | 0.515 |
